# Social Isolation, Resilience, and Psychological Well-Being in Older Chinese Americans

**DOI:** 10.1093/geroni/igab046.1135

**Published:** 2021-12-17

**Authors:** Xiang Qi, Wei Zhang, Katherine Wang, Yaolin Pei, Bei Wu

**Affiliations:** 1 Rory Meyers College of Nursing, New York, New York, United States; 2 University of Hawaii at manoa, Honolulu, Hawaii, United States; 3 Duke University, Durham, North Carolina, United States; 4 New York University, New York, New York, United States

## Abstract

Using data collected in 2018 on 398 older Chinese Americans aged 55+ residing in Hawaii, we examined the associations of social isolation with psychological well-being and the mediating role of resilience. Social isolation was measured by their marital status, living arrangement, contact with children/family/friends, and participation in social activities. Psychological well-being was measured by psychological distress, life satisfaction, and happiness. Results from multivariate linear regressions and ordered logistic regressions showed social isolation was positively associated with psychological distress (β=0.017, p<0.05), and negatively associated with life satisfaction (β=-0.220, p<0.001) and happiness (β=-0.086, p<0.05) . By contrast, resilience was associated with lower psychological distress and higher life satisfaction and happiness. Moreover, mediation analysis showed that resilience contributed to 32% of the association between social isolation and psychological distress, 24.9% of the association between social isolation and life satisfaction, and 16.3% of the association between social isolation and happiness.

